# The Phased Implementation of a National Telehealth Weight Management Program for Veterans: Mixed-Methods Program Evaluation

**DOI:** 10.2196/diabetes.9867

**Published:** 2018-10-09

**Authors:** David E Goodrich, Julie C Lowery, Jennifer A Burns, Caroline R Richardson

**Affiliations:** 1 VA Center for Clinical Management Research, VA Ann Arbor Healthcare System Ann Arbor, MI United States; 2 Department of Family Medicine School of Medicine University of Michigan Ann Arbor, MI United States

**Keywords:** obesity, veterans health, telemedicine, adoption, qualitative research, self-management

## Abstract

**Background:**

The burden of obesity is high among US veterans, yet many face barriers to engaging in in-person, facility-based treatment programs. To improve access to weight-management services, the Veterans Health Administration (VHA) developed *TeleMOVE*, a home-based, 82-day curriculum that utilizes in-home messaging devices to promote weight loss in VHA patients facing barriers to accessing facility-based services.

**Objective:**

The primary aim was to establish preliminary evidence for the program by comparing outcomes for *TeleMOVE* with standard, facility-based MOVE weight-management services (group, individual modalities) over the evaluation period based on the number of patients enrolled per site and the program’s clinical effectiveness, as demonstrated by average weight lost per patient. The secondary aim was to understand factors influencing *TeleMOVE* implementation variability across demonstration sites to develop recommendations to improve national program dissemination.

**Methods:**

We employed a formative mixed-methods design to evaluate the phased implementation of *TeleMOVE* at 9 demonstration sites and compare patient- and site-level measures of program uptake. Data were collected between October 1, 2009 and September 30, 2011. Patient-level program outcomes were extracted from VHA patient care databases to evaluate program enrollment rates and clinical outcomes. To assess preliminary clinical effectiveness, weight loss outcomes for veterans who enrolled in *TeleMOVE* were compared with outcomes for veterans enrolled in standard MOVE! at each demonstration site, as well as with national averages during the first 2 years of program implementation. For the secondary aim, we invited program stakeholders to participate in 2 rounds of semistructured interviews about aspects of *TeleMOVE* implementation processes, site-level contextual factors, and program delivery. Twenty-eight stakeholders participated in audio-recorded interviews.

**Results:**

Although stakeholders at 3 sites declined to be interviewed, objective program uptake was high at 2 sites, delayed-high at 2 sites, and low at 5 sites. At 6 months post enrollment, the mean weight loss was comparable for *TeleMOVE* (n=417) and MOVE! (n=1543) participants at −5.2 lb (SD 14.4) and −5.1 lb (SD 12.2), respectively (*P*=.91). All sites reported high program complexity because *TeleMOVE* required more staff time per participant than MOVE! due to logistical and technical assistance issues related to the devices. High-uptake sites overcame implementation challenges by leveraging communication networks with stakeholders, adapting the program to patient needs whenever possible, setting programmatic goals and monitoring feedback of results, and taking time to reflect and evaluate on delivery to foster incremental delivery improvements, whereas low-uptake sites reported less leadership support and effective communication among stakeholders.

**Conclusions:**

This implementation evaluation of a clinical telehealth program demonstrated the value of partnership-based research in which researchers not only provided operational leaders with feedback regarding the effectiveness of a new program but also relevant feedback into contextual factors related to program implementation to enable adaptations for national deployment efforts.

## Introduction

### Background

In 2016, 42% of the patients receiving care in the Veterans Health Administration (VHA) were obese and 37% were overweight, putting these individuals at risk for obesity-related comorbidity, functional impairment, and diminished quality of life [1,2]. Since 2006, VHA patients have had access to an evidenced-based national weight-management program called MOVE! [1,3]. MOVE! programming relies heavily on group- or individual-based psychoeducational modes of delivery that require patients to visit a facility to receive face-to-face care [4,5]. However, for some veterans seeking phone-based counseling, many facilities have difficulty providing phone-based support due to insufficient staff time or training [6,7]. Furthermore, many patients have difficulty attending on-site programming due to barriers related to logistics (cost, distance, and time), transportation, weather, and conflicts with the scheduled times of available programming [8].

To address these access barriers, the VHA National Center for Health Prevention and Disease Prevention (NCP), which oversees MOVE!, collaborated with the VHA Telehealth Services Home Telehealth (HT) Program to develop a telehealth program called *TeleMOVE*. The goal of this joint effort was to combine MOVE! content with HT’s strengths in deploying innovative health informatics, disease management, and telehealth technologies to overcome barriers to care by delivering coordinated and supportive care management through automated communication protocols [9,10]. *TeleMOVE* was created for delivery via asynchronous in-home messaging devices, which collect and transmit (store and forward) patient data from the patient to a care coordinator at the facility overseeing care. These devices enable clinicians to prospectively monitor and support patient self-management activities more efficiently and allow for a greater number of patients to engage in programming than relying on conventional face-to-face or phone-counseling protocols.

The impetus for rapid *TeleMOVE* implementation was driven by the need for the VHA to increase access to care services for veterans, particularly in rural regions, and less by the evidence for the application of telehealth technology to obesity treatment. The adoption of in-home messaging devices was informed by clinical evidence supporting the benefit of intensive monitoring for weight management through regular engagement in self-weighing, self-guided psychoeducational materials, and helping patients feel accountable to their health care team, particularly through regular brief motivational counseling phone calls with an interventionist such as an HT clinician [11-13].

Although there was evidence for the efficacy of individual voice recognition (IVR) and phone coaching for weight management, studies of in-home messaging devices had not been rigorously evaluated for health promotion application before *TeleMOVE* implementation. VHA policy leaders recognized that the implementation of this untested innovation would benefit from a systematic phased implementation in which VHA researchers employed pragmatic research methods to rapidly and rigorously evaluate the program to identify implementation barriers, assess clinical impact, and develop recommendations to inform national program dissemination efforts [14-16]. National leaders encouraged demonstration sites to adopt the mantra, “Learn, evaluate, and improve,” consistent with the principles of a learning health care system [17].

### Objectives

This implementation evaluation had 2 aims. First, we sought to establish preliminary evidence for the impact of *TeleMOVE* by measuring patient engagement (enrollment numbers), as well as to assess the program’s clinical effectiveness to yield weight loss by comparing weight loss outcomes with similar patients enrolled in existing facility-based MOVE! weight management services at the participating demonstration sites. Second, we examined variability in *TeleMOVE* implementation across demonstration sites using qualitative methods to identify contextual factors that distinguished facilities with high program implementation compared with those with poor indicators of implementation. We hypothesized that sites with higher uptake of *TeleMOVE* would demonstrate greater levels of program enrollment, average weight loss per participant, and theory-based constructs of program implementation relative to low-uptake sites using the Consolidated Framework for Implementation Research (CFIR) [18,19] to assess 39 relevant constructs to the implementation of new interventions at 5 domains or levels of assessment (intervention characteristics; inner and outer setting in which the implementation occurs; characteristics of the individuals involved in the implementation; and the process of implementation itself). Ultimately, the goal of this partnership-based implementation evaluation was for researchers to provide policy leaders with insights into factors affecting implementation and sustainment of the innovation to improve program dissemination efforts throughout VHA.

## Methods

### Study Design

We used a formative mixed-methods design to evaluate implementation of *TeleMOVE* across 9 Veterans Health Administration medical centers (VAMC) over a 2-year period using a parallel in-person MOVE! cohort as a nonrandomized comparison group for quantitative analyses and qualitative interview methods to understand how contextual organizational factors influenced variability in *TeleMOVE* implementation uptake across sites.

### Setting

To facilitate organizational learning, *TeleMOVE!* was implemented in a systematic, multi-phased deployment that is described in Table 1. The first phase began with national operational stakeholders planning the program rollout in the first half of 2009 and recruiting a single VAMC that specialized in telehealth programming to carry out a 3-month single-site demonstration beginning in September 2009. Despite not being the focus of this evaluation, a summary of implementation activities from phase 1 are provided to illustrate the benefit of employing this single site to develop initial implementation toolkit resources to support scale-up and further iterative testing at additional demonstration sites. This study assessed program implementation at 9 VAMCs from 3 VHA regional health networks in the northeastern, southeastern, and middle southern United States that volunteered to participate in phase 2 of the systematic implementation between October 1, 2009 and February 28, 2010. Follow-up data collection at these 9 sites continued until September 30, 2011 to evaluate long-term implementation outcomes.

Phase 2 began in October 2009, with an Web-based training conference about *TeleMOVE.* Patient enrollment began in the first week of November 2009 and continued up to February 2010. Biweekly calls were held between national program leaders and demonstration site personnel to share problems experienced in implementing *TeleMOVE* as well as solution strategies to overcome these challenges. The research team attended these calls to record process notes. All demonstration sites were asked to recruit at least 30 patients during phase 2 and to only use a single model of telemessaging device. In phase 3, the national rollout of the *TeleMOVE* program began in April 2010 and continues to the present.

**Table 1:** Summary of pilot phases of *TeleMOVE* implementation and stakeholders.

Phase		Participants		Implementation activity and evaluation method	
**Planning**					
	March 2009		NCP^a^, TS^b^, and regional network leaders		Invitation for 10 HT^c^ programs to submit written intent to volunteer to pilot *TeleMOVE* Define collaborative roles and responsibilities for NCP/TS Create timeline for phased implementation plan Update implementation plan draft
	July-August 2009		Staff from 1 VAMC^d^ NCP, TS, and regional network leaders		Weekly planning meetings Track challenges and facilitators to *TeleMOVE* during pilot to develop implementation plan Review readiness of cross-training modules for *TeleMOVE* providers
**Phase 1**					
	September-November 2009		1 VAMC Local staff		Enroll 30-45 patients to develop implementation methods and toolkit
**Phase 2 training**					
	October 2009		Staff from 9 VAMCs NCP, TS, and regional network leaders		Share early learnings/challenges from phase 1 site Disseminate program materials Share workflows and administrative procedures
**Phase 2 start**					
	November-February 2010		Staff from 9 VAMCs		Enroll 30-60 patients per medical center Monitor and troubleshoot pilot implementation Identify key learnings; develop solutions to barriers Refine implementation plan for national rollout
**Phase 3**					
	April 2010-September 2011		Interested VA facilities		National goal to enroll 10,000 patients per year Enroll panels of 80-120 patients per medical center Funding for care for up to 300 patients per network

^a^NCP: National Center for Health Promotion and Disease Prevention.

^b^TS: Telehealth Services.

^c^HT: Home Telehealth.

^d^VAMC: Veterans Health Administration medical centers.

### Overview of the MOVE! and TeleMOVE Programs

#### MOVE! Weight Management Services

Standard MOVE! treatment services were implemented throughout VHA in 2006 as a comprehensive, evidence-based lifestyle approach to weight management for veterans [1,3]. In 2010, the core components of MOVE! included annual comprehensive screening for overweight and obesity and brief conversations between patients and clinicians about weight management that included the option for referral to MOVE!. Referred patients then completed a needs assessment called the MOVE!23 Questionnaire that helped clinicians to provide tailored written feedback and handouts on possible areas for lifestyle change. MOVE! featured a behavior-based diet and physical activity self-management support delivered through a variety of modalities that were predominantly facility-based, such as group 71.88% (393,774/547,790) and individual 20.50% (112,299/547,790) face-to-face counseling as well as telephone-based counseling 7.62% (41,717/547,790) [20]. These modalities were delivered by licensed providers from dietetics, nursing, psychology, physical therapy, and social work, utilizing didactic instructions, interactive exercises, and content based around a MOVE! handout booklet. Participants set personal goals with the help of clinicians and were given log sheets and pedometers to monitor behavioral and weight changes. Program staff assessed weights at encounters to monitor the patient’s progress. Programming options varied across facilities due to staffing and space constraints but ranged in intensity from 6 to 12 structured sessions delivered over 4 to 8 weeks, with sessions ranging between 30 to 90 min in duration. In 2010, participants averaged 4.5 visits in MOVE! annually with the majority of visits occurring within 6 months of enrollment [20]. In 2008, VHA began developing *TeleMOVE* as a MOVE! treatment modality that helped patients overcome the barriers to participating in time-specific, facility-based programming via automated, asynchronous telehealth devices that offered patients a flexible and convenient approach to weight management. Notably, *TeleMOVE* excluded services delivered via clinical video telehealth technologies, such as live MOVE! group sessions, simultaneously broadcast to patients at a remote community-based outpatient clinic (CBOC).

#### Telehealth Device

*TeleMOVE* was implemented with the Health Buddy, an automated messaging device developed by the Bosch-Health Hero Network (Palo Alto, CA), which enabled daily communication between a *TeleMOVE* coordinator and the participant. The messaging device was the size of a clock radio and featured 4 buttons, a liquid crystal display screen, a speaker, and a connection to a landline phone. *TeleMOVE* utilized a disease management protocol (DMP) that featured daily communications based on a series of algorithmic interview questions delivered to patients in their homes via a display on the device that assessed patients’ symptoms, health factors, educational needs, and self-management status. In the course of these interactive dialogues with the Health Buddy, patients entered weight information and any responses to daily prompts to be forwarded via landline phone each night to a vendor server from where it was then forwarded to a *TeleMOVE* coordinator for review.

#### Intervention Program

Upon completing MOVE! enrollment activities, patients who elected to choose the *TeleMOVE* programming modality received a Health Buddy, MOVE! handout booklet, a pedometer to track daily ambulatory activity (steps), and a digital scale to use at home. The participant and a *TeleMOVE* coordinator then used the MOVE! booklet to develop a patient-centered treatment plan with specific weight and behavioral change goals to monitor progress. Once this plan was agreed upon, it was recorded in the patient’s medical record. Following the installment of their device, a patient would commence participation in 82 daily communications or sessions at an agreed upon time. The DMP engaged the participant at their home, in a 5-min interactive educational module that was displayed on the device screen. These modules covered topics pertinent to weight management, such as nutrition, exercise, behavior modification, self-monitoring, and goal setting, adapted from the content used in the standard MOVE! booklet for in-person programming for individuals and groups. At the end of each module, the participant was prompted to answer a series of multiple-choice questions to evaluate user understanding. Correct answers were reinforced with positive affirmations and for incorrect responses, participants were encouraged to reference modules from the accompanying MOVE! booklet.

The Health Buddy prompted participants to provide daily weight readings from their digital scale to encourage tracking of weight management progress. If a participant went 30 days or longer without losing half to 2 lb per week or lost weight too quickly, a trigger alert for re-evaluation would occur. Participants received 10- to 20-min telephone calls from a *TeleMOVE* coordinator every 30 days that had the purpose of re-evaluating patient goals while providing motivational and problem-solving support. Coordinators also called participants for affirmative responses to *red alert* questions about increased pain or emotional distress. Finally, the 82 modules had to be completed within 90 days, at which point the participant would decide if they would like to repeat the program for a second cycle. Participants could pause the program for breaks up to a total of 7 days in case of acute illnesses or vacations. Although program duration spanned nearly 3 months, the total dose of patient participation was designed to be roughly equivalent to traditional clinician-delivered MOVE! modalities.

#### TeleMOVE Interventionists

The *TeleMOVE* implementation guide permitted the program to be administered flexibly either by the MOVE! or HT programs at each facility or a combination of both services. While most VHA telehealth programs were typically staffed by registered nurses, nurse practitioners, or social workers, the addition of the weight management DMP permitted other disciplines (eg, registered dietitians/nutritionists and psychologists) to deliver *TeleMOVE* to veterans, provided they agreed to complete local, regional network, and national training related to MOVE! and HT competencies. *TeleMOVE* participant materials were often distributed to patients through each hospital’s prosthetics service. Given the potential cross-disciplinary staffing complexities of *TeleMOVE*, the implementation guide provided detailed guidance regarding the methods to track workload, coordinate the distribution of program materials, and estimate staffing resources to meet projected needs of the facility’s patient population.

### Quantitative Evaluation

#### TeleMOVE Participants

The quantitative aspect of this mixed-methods study evaluated 2 cohorts of VHA patients who enrolled in either *TeleMOVE* or standard in-person MOVE! programming during fiscal years (FYs) 2010 and 2011 (October 1, 2009 to September 30, 2011) at each of the 9 demonstration sites. The in-person MOVE! cohort served as a parallel, nonrandomized comparison group to evaluate preliminary clinical outcomes for *TeleMOVE.* All VHA weight management program enrollment occurred through each site’s MOVE! program, where each patient was required to complete a 23-item questionnaire called the MOVE!23 about their weight history [3,21] and to consult with a MOVE! clinician to discuss programming options before starting the treatment. Veterans eligible for MOVE! were those who were obese (body mass index, BMI ≥30) or who were overweight (25≤BMI<30) with a weight-related health problem (diabetes, hypertension, degenerative joint disease, dyslipidemia, obstructive sleep apnea, or metabolic syndrome) [3]. Veterans could choose to enroll in standard MOVE! services or *TeleMOVE.* To enroll in *TeleMOVE*, patients had to meet additional criteria including not being enrolled in another HT program (eg, for noninstitutional care, acute care management, or chronic care management); having a working landline telephone; and having no plans to relocate during the 6 months of the initial enrollment in *Tele* *MOVE*. Patients enrolled in these program modalities were identified retrospectively using VHA Decision Support System identifier/stop codes to capture workload credit from administrative databases.

#### Quantitative Data Extraction and Analysis

Quantitative data was extracted from VHA patient care databases to describe patient characteristics, program use, and weight changes associated with program participation at each of the 9 demonstration sites. Participant demographic characteristics and program utilization data were extracted from the VHA Service Support Center-hosted visits ProClarity cube. Data pertaining to medical comorbidities and change in weight were extracted from the VHA Corporate Data Warehouse (CDW). Program use was characterized by 2 indicators: program enrollment and program engagement. Distinctions in program *enrollment* versus engaged *participation* were based on operational definitions developed by NCP [20]. Enrolled patients were required to have at least one visit within 180 days of the date of enrollment. As an indicator of more sustained program use, *engaged* participation was defined as patients having more than 2 visits within 180 days of enrollment. The primary quantitative outcomes for this evaluation were (1) cumulative number of patients *engaged* in *TeleMOVE* and MOVE! in FY 2010 and FY 2011 at each demonstration site defined by having greater than 2 visits over 180 days; (2) mean weight loss per patient achieved after 6 months of program participation; and (3) the percentage of participants with clinically meaningful weight loss (≥5% body weight from enrollment to 6-month follow-up) [22]. Baseline weight was determined by extracting the closest clinical weight measure within ±30 days of enrollment from vital status files in CDW. Follow-up weights were assessed at 180 days from enrollment using a 60-day window before and after the 180-day increment [20]. To provide a basis to interpret preliminary *TeleMOVE* effectiveness, we provided MOVE! statistics for FY 2010 to serve as nonstatistical comparator references for key weight loss outcomes, including average *national* mean values for weight loss, percentage of weight loss, change in BMI, and the proportion of participants achieving clinically significant weight loss (≥5% of pretreatment weight) [23].

Indicators of site implementation effectiveness were rated based on attaining targets for program enrollment *and* attaining average weight loss of at least one pound for program participants. For phase 2 pilot implementation, we assessed whether each demonstration site could enroll at least 30 patients over 4 months as an indicator of implementation effectiveness. As indicators of sustained implementation effectiveness, we evaluated whether each demonstration site could accumulate patient panels of at least 100 patients and attain average weight loss per participant by the end of FYs 2010 to 2011. Classification as a *high-uptake* site was based on attaining NCP/HT enrollment targets while also attaining an average weight loss per participant. *Low*-*uptake* sites were categorized by attaining only 1 or no indicators of program effectiveness (ie, low enrollment/high clinical effectiveness, high enrollment/low clinical effectiveness, or low enrollment/low clinical effectiveness). These program indicators were tracked over 2 years to assess the sustainability of early program adoption as well as to assess which contextual factors (as identified by the qualitative data) were correlated with program effectiveness to identify possible determinants of successful implementation. Descriptive statistics were used to summarize patient characteristics and program utilization patterns. Paired *t* tests were used to compare patient characteristics and change in weight status from baseline, adjusting for clustering by site. Quantitative data analysis was performed using SAS Version 9.2 (SAS Institute Inc, Cary, NC).

### Qualitative Evaluation

#### Qualitative Stakeholder Interviews

The evaluation plan called for conducting 2 rounds of semistructured interviews at each of the 9 phase 2 demonstration sites. The first round of interviews was conducted by phone, 3 to 6 months after phase 2 (June-August 2010) and the second round of in-person interviews were conducted 6 months after the start of phase 3 (November 2010-April 2011) to capture the dynamic nature of the implementation process. Key facility- and regional-level managers and program staff involved in *TeleMOVE* implementation were invited by email to participate in the interviews, including *TeleMOVE* care coordinators, HT directors, MOVE! coordinators, MOVE! dietitians, physician program champions, program support assistants, regional data analysts, and regional network program coordinators. Participation in interviews was voluntary, and we asked for additional names to ensure that we invited all individuals involved in *TeleMOVE.*

Verbal consent and permission to digitally audio-record interviews were obtained from participants at the start of their first interview. Staff at 3 sites declined to participate in both rounds of interviews. A total of 42 VHA stakeholders were invited to participate in an interview, and 66% (28/42) agreed to participate in at least one interview; 22 participated in the phone interviews, and 21 participated in on-site interviews. Interview ranged from 18 to 86 min in duration and was digitally audio-recorded and transcribed verbatim into Microsoft Word documents. Additionally, call minutes from biweekly conference calls held among the demonstration sites during the evaluation were analyzed to understand contextual factors affecting implementation effectiveness.

#### Qualitative Data Collection, Coding, and Analysis

The CFIR [18] was used to develop the 2 interview guides and offered a framework for qualitative coding and of those contextual factors that could affect implementation success [17,21,22]. See Multimedia Appendices 1 and 2 for the interview guides. The CFIR organizes 39 constructs that influence implementation into 5 major domains: intervention characteristics, inner setting, outer setting, characteristics of the individuals involved in the implementation, and the process by which implementation is accomplished. Abbreviated definitions of CFIR constructs and domains are located in additional file 3 [24]. Interview transcripts were coded deductively using a codebook based on the CFIR and a descriptive content coding approach [19]. QSR International’s NVivo software version 10 was used to facilitate coding. Each interview transcript was independently reviewed and coded by at least 2 members of the research team and a fourth coder helped achieve consensus in cases of disagreement (JCL) [25]. Codes were compared, and differences were resolved by a consensus discussion. A memo was created for each site to summarize the top 10 CFIR constructs mentioned by respondents that were strongly associated (positively or negatively) with implementation outcomes (enrollment process and weight loss). Memos were compared across sites to identify the constructs that were most consistently associated with high or low uptake of the intervention across sites. A fifth team member (CRR) performed a member check to verify the validity of the top themes identified by the team.

### Human Subjects’ Protection

This research study was approved by the VA Ann Arbor Healthcare System Institutional Review Board (2010-010042) with a waiver of signed informed consent for staff interviews and for secondary data analysis of deidentified patient-level outcome data.

## Results

### Quantitative Results

**Figure 1:**
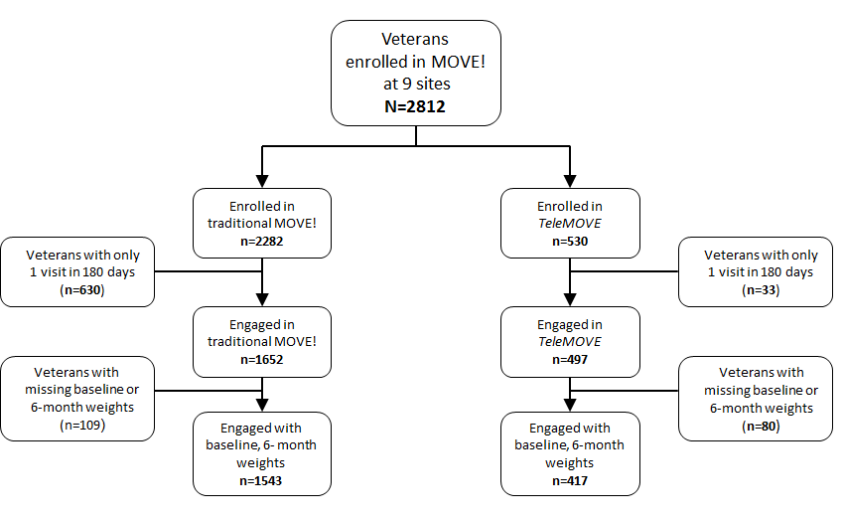
Comparison of enrollment in evaluation cohorts for year 1 of implementation.

Figure 1 presents a comparison of enrollment in *TeleMOVE* and standard in-person MOVE! at the 9 demonstration sites during the first year of program implementation. Notably, among patients who enrolled and engaged in *TeleMOVE*, 93.9% (467/497) engaged in 2 or more visits over 6 months compared with 71.97% (1189/1652) for MOVE!.

**Figure 2:**
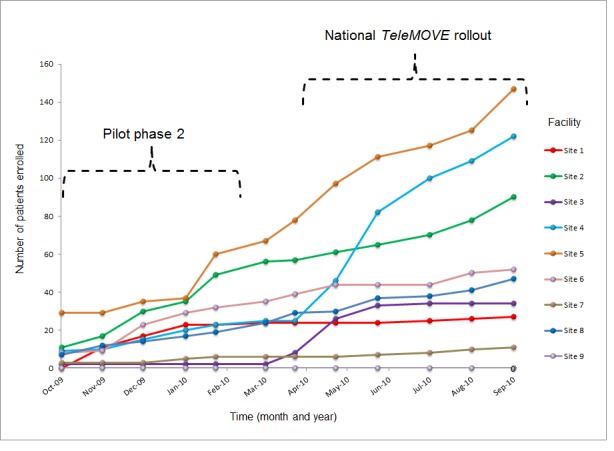
Cumulative enrollment across sites during year 1 of *TeleMOVE* implementation.

Figure 2 shows cumulative enrollment in *TeleMOVE* during the first year of implementation. Moreover, 3 sites attained the phase 2 goal of enrolling 30 or more patients, 3 sites enrolled less than 30 (mean=25), and 3 sites recruited between 0 and 6 patients. Following phase 2, 2 of the 9 sites attained cumulative enrollment levels of at least 100 patients by the end of FY 2010, and 4 sites attained this target in FY 2011.

There were significant differences in demographic characteristics between those who chose to enroll in each program modality (see Table 2)*.* Both Hispanics and African Americans were more likely to choose in-person MOVE! than *TeleMOVE*, whereas there were no differences in enrollment based on sex*. TeleMOVE* enrollees were also older than those who chose in-person MOVE!, whereas rural veterans were more likely to choose *TeleMOVE* than urban veterans. Finally, *TeleMOVE* participants were significantly heavier than in-person MOVE! participants. However, there were no differences between program participants with respect to the burden of medical comorbidities as measured by the Charlson Comorbidity Index [26].

Preliminary indicators of clinical effectiveness are summarized in Table 3. The average weight loss for both *TeleMOVE!* and standard MOVE! was at −5.2 lb (SD 14.4) and −5.1 lb (SD 12.2), respectively, with no statistically significant differences between the 2 delivery modalities (*P*=.91). At the demonstration sites, both program modalities slightly outperformed average national in-person MOVE! results with respect to weight loss. These preliminary weight loss outcomes suggest that the number of patients needed to treat by *TeleMOVE* to achieve clinically meaningful weight loss (≥5%) is 5, which is indicative of a highly effective treatment.

Multimedia Appendix 3 presents site-level indicators of *TeleMOVE* implementation. Although there was significant variability in baseline characteristics of demonstration sites with respect to patients served, rurality, and proportion of patients served older than 55 years, no discernible association could be made with these facility characteristics and indicators of enrollment and clinical effectiveness below. The table combines enrollment and weight loss outcomes to generate a combined indicator of implementation effectiveness. *High-uptake* sites 4 and 5 not only achieved phase 2 enrollment targets of ≥30 patients but also demonstrated the ability to sustain higher patient panel sizes during national rollout while providing clinical benefit. Sites 6 and 7 exemplified cases of *delayed* high-uptake in which effectiveness not was manifested until year 2, when enrollment numbers increased.

*Low-uptake* sites were broadly characterized by low enrollment at sites 1, 3, 8, and 9. However, site 2 was a low-uptake site that had high enrollment numbers without achieving meaningful weight loss outcomes. Notably, both sites 1 and 9 had low enrollment rates and displayed declines in weight loss outcomes.

**Table 2:** Demographic characteristics of engaged *TeleMOVE* and MOVE! year 1 participants.

Characteristic		*TeleMOVE* (n=497)	MOVE!^a^ (n=1648)	*P* value^b^
Age in years, mean (SD)		57 (9.5)	55 (11.0)	<.001
Male, n (%)		422 (84.9)	1434 (87.01)	.23
**Race^b^, n (%)**				<.001
	White	301 (80.1)	853 (65.31)	
	Black	60 (16.1)	413 (31.62)	
	Other	11 (3.1)	40 (2.96)	
Ethnicity^c^ (Hispanic), n (%)		7 (1.7)	66 (4.88)	.006
Rural address, n (%)		287 (57.9)	691 (41.93)	<.001
Baseline (lb), mean (SD)		256 (51)	243 (49)	<.001
Baseline body mass index, mean (SD)		37.5 (6.9)	35.5 (6.3)	<.001
Charlson score, mean (SD)		1.7 (1.9)	1.6 (1.9)	.39

^a^Excludes patients enrolled in *TeleMOVE* during the same time period.

^b^Paired *t* test comparisons of patient characteristics were adjusted for clustering by site.

^c^Available data to calculate % race/ethnicity variables were *TeleMOVE* (N=402) and MOVE! (N=1353).

**Table 3:** Comparison of weight change outcomes in year 1 for engaged participants.

Characteristics	National MOVE! cohort (N=31,854) fiscal year 10^a^	MOVE! participants (N=1553)	*TeleMOVE* participants (N=417)	*P* value (MOVE! versus *TeleMOVE*^b^)
Six-month weight (lb), mean (SD)	−3.6 (0.1)^c^	−5.13 (12.4)	−5.22 (12.4)	.90
Six-month change (BMI^d^), mean (SD)	−0.5 (0.0)^c^	−0.75 (1.8)	−0.70 (2.4)	.72
Change in body weight, n (%)	−1.4 (0.1)^c^	−2.02 (5.0)	−2.01 (5.6)	.95
Number of patients with >5% weight loss, n (%)	5925 (18.60)	372 (24.11)	92 (22.1)	.31

^a^Normative in-person averages from national FY 2010 MOVE! report [23].

^b^Paired *t* test comparisons were adjusted for clustering by site.

^c^Note, all fiscal year 10 national *MOVE!* statistics used SEs and not SD.

^d^BMI: body mass index.

### Qualitative Findings

Among the 6 sites that participated in qualitative interviews, we identified 5 CFIR constructs that illustrated key contextual factors that distinguished high from low implementation sites: complexity, patient needs and resources, networks and communications, leadership engagement, and reflecting and evaluating. Table 4 provides illustrative quotes for each construct. Multimedia Appendix 4 provides a detailed chronological summary of formative findings shared with operational partners over the course of the 2-year evaluation.

#### Complexity

Complexity refers to the perceived difficulty of implementing an intervention especially with respect to the duration, scope, disruptiveness, and intricacy of the steps involved. *TeleMOVE* was viewed as complex across all sites because it required (1) several time-consuming and intricate logistical steps simply to enroll a patient, check devices, and mail the messaging device and program materials; (2) troubleshooting installation and resolving ongoing device issues with patients; and (3) resolving issues related to the messaging devices not being user-friendly for older patients or accessible for veterans without a landline phone.

Across all 6 sites, respondents felt that although *TeleMOVE* was a progress toward helping veterans access weight management services in their homes, the technical compatibility of the telehealth device with patients’ home connectivity was an increasing barrier as telecommunications companies upgraded veterans’ landline homes to digital technology. Many patients who were interested in *TeleMOVE* discovered that they no longer had a home landline phone to hook up the telehealth device because they relied primarily on either cellular or digital cable phone services.

**Table 4:** Qualitative data illustrating contextual factors distinguishing *TeleMOVE* uptake.

CFIR^a^ construct	High-uptake site	Low-uptake site
Complexity	*Making sure your clinics are started up right, making sure you’ve got everything in place before you get, take on your first patient, knowing how to enroll, knowing how to do the forms correctly, knowing how everything needs to run, getting the equipment to the patient, getting the program itself started...* *If I get them [patients], I have them face to face; I have the screening right there...So we’ve eliminated the phone tag and they’re right on board. So, when they get their equipment, I call them the next week. Most of them are already hooked up on the equipment.*	*There were all the hurdles in the world you could imagine. So the six months before [that?] I spent trying to contact everybody and their cousin trying to figure out how to make this happen, how to get the technology...just logistics, you know. How were the pedometers going to be issued turned into a big you know, issue.*
Patient needs and resources	*...They always at least always get a phone call at least once a month even if they did fine,...because they like that accountability...the ones that participate...They like something that will help keep them focused. If they could come see me every day, they might do that. So, this is a way to keep them at home and keep them focused with the weight loss.*	*I would put more people on the Tele-Move program if I could but a lot of people do not have computers or land lines. All they have is cell phones.* *...I would like to see...more initial face-to-face um, contact with the Veteran before they’re actually in the program and issued the equipment...If we were able to arrange something like...the care coordinators physically go to those sites at the designated time to meet with the Veterans, to try and do a group enrollment to establish that rapport with that individual, I think you would probably get more buy in...The way that we’re doing it right now is basically over the telephone...there’s none of that face to face personal interaction.*
Networks and communication	*...and every month we do a phone call conference with all our VISN, with all our care Tele-Move coordinators and stuff and keep them updated on anything that comes out even if it comes out beforehand they’re always in the know. I mean I even get phone calls from other VISNs you know, about things you know, because...we’re a little bit ahead of the game so I’m always trying to help and...give out anything that we develop and because there’s no sense in rewriting the, reinventing the wheel when we already got it to help anybody out.*	*Well as a MOVE! coordinator and being on the calls I tried to solicit you know, the clinic space and the clinic profiles...I was not clear on the information that this was going to be completely within HT. Initially I thought that the HT Move was going to be within MOVE!...There were a lot of different people trying to you know, coordinate the program...But we were some time in before we clearly understood that all this stuff was going to be within HT you know.*
Leadership engagement	*You know, they [facility leaders and managers] were really supportive and they always wanted to know what was going on you know, what kind of data and stuff we were getting.*	*Um no, leadership—I never felt supported at all for any of home Tele-Health with leadership at this hospital. With my immediate supervisor, yes but with senior management no...*
Reflecting and evaluating	*This is what I send to CCHT Director every month is the non-responders because that’s what my quality improvement deal is...I’m at average monthly compliance of 58, my goal is 80. Well every month we do a report...our chief has us do it; We have to do a weight loss report and weight gain percentage and all that kind of stuff...we get from our data warehouse and…we can pull triglycerides, HDL, blood pressures it kind of depends on whatever we want to be tracking. I can...see like where they were when they started until now to kind of see if there has been improvement on those ends...we always kind of we look at our numbers and stuff making sure. And we do monthly calls just here at our facility you know, to make sure we’re all on the same page and not doing something completely different than the other...We’re always trying to improve and do better.* *It really has been good to go through the pilot and get a lot of feedback from the CCHT Coordinator because we were able to kind of stream line a lot of things.*	*...I don’t write down or have any tracking system for how the patients are doing um, but I think we have few enough patients where I can almost just remember how each one is doing, at least of my patients.* *I haven’t seen that [program feedback data] but I haven’t asked for it either you know, so. I mean because I’m not supervising that...*

^a^CFIR: Consolidated Framework for Implementation Research.

Although many patients were pleased with the telehealth devices, some patients were frustrated by connectivity issues and others were disappointed with the device’s simple interface. Often, these dissatisfied patients chose not to use their device, thereby reducing program productivity due to the time needed for program coordinators to contact these patients, return the devices back to the VA hospital, and to repurpose the device for another patient.

Coordinators across sites spent considerable time in calling patients to provide technical assistance, particularly for older veterans who were less confident in using the telehealth devices despite the relatively simple device interface. Coordinators also noted the need to address the ongoing issue of false alerts for issues such as low self-reported mood or errors in the transmission of weight data due to issues with the interface of the digital scales with the Health Buddy. Many of these complexity issues stemmed from the fact that most sites initially attempted to conduct screening and enrollment over the phone rather than conducting face-to-face orientations. With thoughtful experimentation, high-uptake sites found better ways during the phased implementation to mitigate complexity barriers by proactively preparing patients for the device use, whereas low-uptake sites continued to struggle with these issues without re-examining their workflow to identify areas to mitigate problems.

Low-uptake sites also found the *TeleMOVE* implementation complex due to unanticipated issues with the device platform and adopting new administrative procedures. Meeting minutes from phase 2 implementation conference calls among demonstration sites revealed that the requirement to administer *TeleMOVE* using Health Buddy devices was particularly disruptive to sites 7, 8, and 9 that relied primarily on Viterion 100 messaging devices for HT programming (Viterion TeleHealthcare, LLC; Tarrytown, NY). The subsequent process to adopt another HT platform to implement *TeleMOVE* resulted in long delays. Additionally, low-uptake sites reported the process to adopt a new interdisciplinary method to assign workload credits among HT and MOVE! staff and have these changes approved by regional leadership slowed program uptake.

#### Patient Needs and Resources

This construct reflects the extent to which patients’ needs, as well as barriers and facilitators to these needs are accurately known and prioritized by program staff. Accurately assessing patients’ home connectivity was a critical component of the recruitment and enrollment process. Over time, higher performing coordinators developed in-person protocols that carefully assessed this issue during screening and enrollment, recording declined patients’ names in a file for contact when *TeleMOVE* moved to more flexible platforms such as mobile phones, individual voice recognition, or the internet. Staff at high-uptake sites utilized these in-person orientations to explain how to use the messaging devices, set initial personalized program goals, and to anticipate requests for technical assistance or follow-up from patients. In comparison, less effective enrollment procedures at low-uptake sites caused the staff to focus much of their attention on enrollment and reacting to technical assistance requests.

High-uptake sites were also more likely to report to patient needs by resourcing *TeleMOVE* with coordinators who possessed health-coaching expertise, which they utilized on both incidental and planned monthly action-planning calls to address patient problems, set goals, coordinate reported health issues with the patient’s provider, and maintain patient engagement and motivation. In contrast, lower-uptake site respondents were less likely to mention personalized contacts to help patients feel engaged, accountable, and motivated. Coordinators across high and low uptake sites also noted that the criterion that veterans only participate in one HT program at a time was counterproductive, since many eligible *TeleMOVE* patients had obesity-related comorbidities such as diabetes, high blood pressure, or high cholesterol and could have benefited from concurrent participation in other HT programs addressing these risk factors in an integrated fashion. Finally, respondents across both high and low uptake sites observed that although patients were prompted regularly by their telehealth devices to provide program satisfaction feedback, vendors refused to share this information with VHA. This contractual dispute denied providers with regular and consistent ratings of program satisfaction that could help inform efforts to make the program more patient centered.

#### Networks and Communications

Strong formal and informal social networks of program staff and leaders were essential to effectively implement *TeleMOVE*. Failure to communicate effectively across services was more likely in low-uptake sites. In contrast, high-performing sites reported a high degree of cooperation between providers across programs and the ability to reach out to other services, providers, and leaders to gain support in planning and delivering the *TeleMOVE* program. One high-uptake site exhibited uniquely strong care management and coordination networks by consulting relevant providers to adjust patients’ medications for blood pressure, cholesterol, diabetes, and psychiatric self-management in response to desirable reductions in weight and lifestyle improvements.

#### Leadership Engagement

The presence of strong commitment and support by leaders is an indicator of an organization’s commitment to implementing an intervention. In contrast to low-uptake sites, among high-uptake sites, there was strong support by hospital leaders as well as by frontline supervisors and midlevel managers overseeing HT and MOVE! programming. This support was manifested by quick approvals for changes in workflows, staffing credit, and resources, with a shared consensus that *TeleMOVE* was viewed as a long-term addition to HT services. At low-performing sites, there was less shared consensus about the benefits of *TeleMOVE* across stakeholders, and facility-level leadership was frequently unaware of the existence of the program.

#### Reflecting and Evaluating

Effective implementations require the ability to regularly reflect and evaluate both quantitative and qualitative feedback regarding the progress and quality of an intervention implementation. This CFIR construct reflects a quality improvement mindset that was present to some degree in all high-uptake sites but absent at the low-uptake sites. High-uptake sites provided specific examples of monitoring various aspects of program implementation and then using these data to identify opportunities to improve care delivery and patient outcomes. Conversely, examples of reflecting and evaluating were largely absent at lower performing sites where program staff were more reactive and less innovative in identifying solutions to issues in implementing the program.

## Discussion

### Principal Findings

Our mixed-methods findings provide preliminary evidence for the clinical effectiveness of the *TeleMOVE* weight-management program for helping veterans lose weight while identifying key characteristics of program users and contextual factors for consideration in scale-up of the intervention at other VHA facilities. This evaluation study is important because it addresses 2 major reasons cited for the low use of mHealth and telemedicine programs in medical settings on a population level: (1) insufficient evidence for the efficacy of the telemedicine innovation and (2) poor insights into how organizational and social contextual factors influence the adoption and routine use of such new technologies [27,28]. This study underscores the benefit of partnerships between researchers and operational programs to rapidly evaluate promising technology innovations in medical settings to help ensure their widespread adoption and sustainability.

Our quantitative findings helped build a case for further adoption of *TeleMOVE* throughout VHA by demonstrating the clinical benefit of the program and by providing VA leaders with insights into likely program users. New telehealth programs are often given low relative priority for adoption [18,29] by health care leaders because it is unclear whether a new program is effective or provides a solution to a key clinical need or issue [27]. However, *TeleMOVE* simultaneously addressed the burden of obesity among veterans and access to weight management treatment services by leveraging existing HT infrastructure to provide a solution to these clinical priorities. Across the 9 demonstration sites, veterans averaged a 5.2 lb (2.4 kg) weight loss over 6 months using *TeleMOVE*, which was comparable with results achieved in standard in-person, facility-based MOVE! programming. These results have since been replicated in subsequent observational studies [30,31]. We also found that *TeleMOVE* users were more likely to be white, older, heavier, and living in rural areas than traditional *MOVE!* program participants. These characteristics are relevant to VHA clinical leaders because it is national VHA priority to reach the 25% of veterans who live in rural areas [32] with preventive services, and higher BMI levels among *TeleMOVE* participants indicate the program reaches patients likely to benefit from weight loss support. Enrollment figures also suggest that it was feasible for the majority of demonstration sites to achieve patient panel sizes in the 80-120 patient range.

This evaluation benefited from the systematic assessment of stakeholders’ perspectives regarding *TeleMOVE!* to identify contextual factors facilitating or impeding program uptake so that refinements to the implementation plan could be made in a timely manner. Notably, stakeholders observed that *TeleMOVE* was a logistically and technically complex intervention that did not result in decreased staff time per patient. Reflecting evaluations of similar telehealth programs [28,33-35], policy makers underestimated the time coordinators and program staff had to take to screen, enroll, and activate patients, including the considerable time for mailing devices, advising on installation, responding to red flag alerts, attempting to reach patients to answer questions, and reacquiring equipment from nonresponders. These logistical challenges reduced staff time for recruitment and enrollment as well as efforts to improve direct contacts with the patient during program participation, suggesting the need for a more cost-effective and user-friendly intervention delivery platform. Requiring demonstration sites to use only the Health Buddy messaging also revealed a key lesson for national implementation: make the *TeleMOVE* DMP interoperable across the different vendor devices to avoid delays in switching platforms.

Stakeholders told us that it is important to consider what the CFIR identifies as outer setting factors interacting with the *TeleMOVE* implementation, including understanding the needs, resources, and circumstances of patients using the program. *TeleMOVE* was implemented during a period when home connectivity was rapidly changing from landline phones to digital and cellular forms of connectivity [36,37]. Many eligible patients were turned away because they lacked a landline phone. Installation and use of the devices was intimidating for some older users while many potential participants had to be turned away because their household no longer had landline telephone access. Although some staff at some sites tried to enroll patients over the phone, face-to-face enrollment was essential to confirm patient expectations about the program and to identify patients most likely to engage in sustained participation over time as has been reported from prior VHA evaluations of telehealth programs for older veterans with chronic disease management needs [34,38]. As mHealth and eHealth interventions continue to advance at a rapid pace, it will remain essential to match patients to a user-friendly intervention platform and to ensure that patients understand how to engage with the intervention platform to attain clinical benefit.

Prior telehealth studies point toward leverage points to improve *TeleMOVE* over time to tailor the program to specific patient groups. Studies at this time reflect our results that those who were less likely to engage in the program were women, younger veterans, and those living in urban areas who may have preferred other telehealth platforms delivered by IVR or mobile phone apps [28,34,36]. These alternative platforms for *TeleMOVE* also reduce some of the logistical barriers that diminish the efficiency of relying on the in-home messaging devices and could allow clinicians at CBOCs in rural areas to enroll veterans rather than having enrollment controlled by program staff at a distant VA facility where HT services are centralized. However, high-uptake sites adopted best telehealth practices to reduce logistical issues and increase patient engagement for older patients by using individual or group enrollment sessions to ensure users understood the DMP protocol, how to use and connect the telehealth device, and solve issues [34]. In addition, despite the automated nature of the DMP, high-uptake sites made a concerted effort to employ coordinators trained in motivational interviewing [39] and theory-based, patient-centered cognitive behavioral change strategies (eg, values clarification for goal setting, problem solving, personalized feedback, self-monitoring, and relapse prevention) to keep patients engaged through regular phone or in-person contacts [40-44].

Interviews also revealed how *TeleMOVE* implementation interacted at multiple levels within demonstration sites’ inner setting. Notably, while demonstration sites already had robust HT programs and saw a value to volunteering for the phase 2 implementation, *TeleMOVE* caused stakeholders to interact with other services in new ways that were disruptive and challenging in some cases. For example, the process of staffing the program and adopting new billing codes to capture workload credits for low-uptake sites was slow and frustrating at sites with poor communication networks between services. Coordination of programming was further complicated by the fact that *TeleMOVE* was a stand-alone platform in which program data was maintained on a separate vendor database. Accordingly, data was not readily accessible to stakeholders (patients or providers) or integrated into the electronic medical record in a usable form [45]. This lack of interoperability from vendor databases to end users such as patients, providers, and program coordinators inhibits utilization for developing goals and monitoring results to make program improvements. High-uptake sites were characterized by coordinators who made an extra effort to organize program data to communicate to program stakeholders, to set performance goals, monitor progress, and share results with the patient, providers, and facility leaders to increase the perceived value of *TeleMOVE* for veteran care.

### Limitations

This study is not without limitations. Notably, the study was a nonrandomized program evaluation of volunteer facilities to the implementation of clinical care program for an older generation of telehealth intervention platform. Generalizability was limited to a small number of sites and clinical stakeholders over a short period, and stakeholders at 3 sites with implementation challenges were unwilling to be interviewed regarding the specific barriers to implementing *TeleMOVE* at their site despite their initial enthusiasm to participate in the early phases of the implementation. Hence, results may not acknowledge significant organizational and contextual factors that may impede effective implementation at a health care facility despite enthusiastic support among some stakeholders. Quantitative analyses of program effectiveness were also limited in that the original design of the study did not call for cost-effectiveness analyses of *TeleMOVE* relative to standard *MOVE!* programming. Such analyses would be difficult because a number of indicators to program effectiveness are not readily accessible from vendor databases such as types of contacts between patients and *TeleMOVE* coordinators, frequency of patient use, reliability indicators (eg, calls for troubleshooting device problems), and patient satisfaction data [46]. Telehealth interventions represent a significant investment, and it is particularly important to determine if devices and programs are user-friendly, well designed, and achieve clinically significant changes in clinical outcomes [46]. Currently, it is difficult to extract *TeleMOVE* data from VHA administrative databases to perform measurement-based care, monitor program process over time, and allow policy makers to make strategic funding decisions. Finally, results from this study may not generalize to other community-based settings that lack the integration of the VHA health care setting and the significant investment in telemedicine platforms.

### Modifications to National Implementation Strategies

The formative nature of this phased implementation program evaluation enabled operational decision makers to obtain real-time feedback from VHA implementation researchers to make several significant modifications to the program implementation guide and toolkit. Below are the recommendations made to operational leaders to inform national implementation efforts that were derived from our mixed-methods evaluation:

- Conduct initial face-to-face screenings and enrollment sessions to assess patient ability and motivation, verify home connectivity status, and proactively address technical questions related to device use and installation.
- Ensure telehealth devices from multiple vendors could all work from the same basic *TeleMOVE* DMP.
- Allow patients to enroll in another HT DMP while in *TeleMOVE* to concurrently address weight-related comorbidities (eg, pain and diabetes).
- Revise implementation guides to emphasize the need for interservice care agreements between facilities MOVE! and HT services to answer and address specific implementation decisions regarding staffing, referrals, panel sizes, workload credit, staffing and funding needs, and procuring and mailing the telehealth and peripheral devices (scales and pedometers).
- Advocate local coordinators to assess staff competencies and encourage staff to undergo recommended standardized trainings in motivational interviewing.

These program modifications were incorporated into the implementation plan used in the subsequent national *TeleMOVE* rollout. Some recommendations could not be easily addressed. For example, staff recommended providing advanced training to HT staff in motivational counseling and in behavioral weight loss strategies. This increased level of training was not incorporated into the national implementation plan, but staff were encouraged to consult with local health behavior coordinators for assistance in these areas. There were also strong recommendations for the development of IVR software version of *TeleMOVE* to address the high number of interested patients without landline phones, but development of such a program was beyond the scope of the project. Finally, it has proven difficult to seamlessly update participants’ medical records with progress data from online vendor monitoring databases.

Although the telehealth technology highlighted by this mixed-methods program evaluation may seem dated by today’s standards, the barriers to implementation of new generations of eHealth and mHealth technologies largely remain the same [47]. Specifically, many technology intervention programs developed by researchers and commercial vendors face challenges with respect to interoperability with the electronic medical records and information networks of most health care providers [48,49]. Most interventions that do provide clinicians and patients with relevant data that inform clinical care decisions are likely unsustainable [50]. Furthermore, the adoption of new mHealth and eHealth technologies occur within an organizational environment in which contextual factors decide whether a promising technology is deployed and sustained over time in an organization and with technology users [51,52]. The present implementation evaluation provided VHA policy makers with proactive feedback on the limitations of *TeleMOVE* and helped clinicians adopt best practices from demonstration sites to help support the existing platform until a newer platform could replace the less efficient telemessaging devices. Consequently, *TeleMOVE* has evolved to meet patient and clinician needs by recently transitioning to an IVR technology to allow patients to use either a landline or cell phone or use a Web browser–based technology that enables patients to use their personal computer or mobile phone (Medtronic Care Management Services, Dublin, Ireland). Although large health systems such as VHA lack the agility to rapidly change technology platforms, *TeleMOVE* exemplifies a case study where VHA’s commitment to systematic, partner-based evaluations of technology implementation efforts has allowed such interventions to spread, evolve, and sustain in the face of dynamic technological environment.

### Conclusions

We showed that an adaptation of telehealth technology could be adapted to promote clinically meaningful weight loss for veterans served by VHA, and formative qualitative data from program stakeholders could help guide national program implementation efforts when summarized by an implementation science framework. Our program evaluation highlights the benefit of implementation researchers partnering with operational initiatives to provide rigorous and rapid evaluation of the systematic deployment of promising innovation. This approach has direct application to the rapid scale-up of promising modes of telemedicine—mHealth and eHealth interventions that have the potential to help provide solutions to gaps in patient care and quality in a dynamic health environment.

## Appendix

Multimedia Appendix 1 Telephone interview guide evaluation of TeleMOVE program implementation.

Multimedia Appendix 2 Site visit follow-up interview guide evaluation of TeleMOVE program implementation.

Multimedia Appendix 3 Site-level indicators of TeleMOVE implementation over 2 years.

Multimedia Appendix 4 Summary of formative outcomes.

## Abbreviations

BMI: body mass index
CBOC: community-based outpatient clinic
CDW: Corporate Data Warehouse
CFIR: Consolidated Framework for Implementation Research
DMP: disease management protocol
FY: fiscal year
HT: Home TeleHealth
IVR: Individual Voice Recognition
NCP: National Center for Health Promotion and Disease Prevention
VA: Veteran Affairs
VAMC: VHA Medical Center
VHA: Veterans Health Administration
