# Supplementary material for: The Phased Implementation of a National Telehealth Weight Management Program for Veterans: Mixed-Methods Program Evaluation
Source: JMIR Diabetes. 2018 Oct 9;3(4):e14. doi: 10.2196/diabetes.9867 (PMC6307696; doi:10.2196/diabetes.9867)
Supplement: Multimedia Appendix 1 [file diabetes_v3i4e14_app1.pdf]

Appendix 1: Telephone Interview Guide
Evaluation of *TeleMOVE* Program Implementation

I. INTRODUCTION

Introduce self and other team members in the room

Hello, my name is [name of interviewer]. I am here with my colleague(s) [name(s) of colleague(s)]. Thank you for agreeing to participate in this interview, we really appreciate your time.

Introduce the project

- As you know, the purpose of this CCHT-Weight Management study is to better understand barriers and facilitators to implementing the CCHT-WM disease manage program that your site encountered while piloting the program.
- We want to take this time to talk to you about your experience, so far, with the *CCHT-WM* program.
- For the purpose of this interview, we will refer to CCHT-WM as *TeleMOVE*.

Explain the purpose of the interview

- Your interview will help us to better understand the challenges and successes of the *TeleMOVE* program at your facility. We will be interviewing multiple people at your facility to gain multiple perspectives. We are really interested in learning more about your own experience with this program.

Describe the audio recording and how we will assure confidentiality and answer any questions

- This interview will be audio taped.
- Please be assured that the tapes and your transcript will be kept confidential. Leadership at your facility, any other co-workers, or NCP or OTS leadership will not have access to any of your responses nor be able to connect your responses to you personally. Only members of our research team will hear the interviews and the audio recording will be destroyed as soon as the transcript is verified and analyzed by the research staff.
- If, at any time, you feel that the questions are too sensitive, I would be happy to turn off the recorder during that portion of questioning. You may also skip any questions you wish during the interview.
- We also wanted to remind you, that we will be giving you a $10 XY gift card as a small token of our appreciation for your participation.

Do you have any questions for me? [Answer any questions]

Are you ready to begin? I'm going to start recording now.

II.BACKGROUND INFORMATION

I am going to ask you a series of open ended questions. I want to hear your thoughts so please do not hesitate to share whatever you believe might be related to any of the topics.

First, I would like to ask you a few questions to help me understand your role in the *TeleMOVE* program.

Appendix 1: Telephone Interview Guide
Evaluation of *TeleMOVE* Program Implementation

Will you please describe your role within your facility? Background Information
PROBES

- What is your title and role within your organization?
- What are your main responsibilities?
- To whom do you report (just position/title, not name)?
- What is your role in the *CCHT or* MOVE! specifically?
- How is your time allocated? FTE?

III. PRE-IMPLEMENTATION EXPERIENCES

I would like to hear more about how the intervention initially started at your site and how you and others viewed the *TeleMOVE* program before it was implemented.

How did your site become involved with this intervention? How did you personally get involved? Intervention Source & Compatibility
PROBES

- How was the decision made to participate in *TeleMOVE*? Who participated in the decision-making process?
- Would you say the implementation of the new *TeleMOVE* was more externally driven, meaning it was a directive from management, or was it internally motivated, meaning the staff at your site felt a need for the program? Why?

What kind of information or evidence were you aware of that showed whether the *TeleMOVE* program would work? Evidence Strength & Quality
PROBES

- Information from your own research, consensus guidelines, published literature, or other sources? From co-workers? From supervisors?
- To what degree did the evidence influence your opinion of *TeleMOVE* before it was implemented?

At the time that your site implemented TeleMOVE, did you see a need for this type of intervention? Why or why not? Patient Needs & Tension for Change
PROBES

- How about other people at your site involved in implementation of *TeleMOVE,* did they feel it was needed? How well do you think *TeleMOVE* meets the needs of patients? (Improved outcomes?)

What kind of care management services were you already offering to patients who are overweight or obese at your site (i.e., by phone, internet, etc.)? Relative Advantage
PROBES

- Please describe the different services/programs.
- To what extent were the existing services multi-disciplinary?
- Were there other alternatives for encouraging patients to engage in physical activity at home?
- Do you perceive the new *TeleMOVE* as being a superior alternative? Why or why not?
- Did you have a previous telemedicine device prior to the current device? If so, how was this earlier device different from the current device?

IV. OPEN-ENDED DESCRIPTION OF IMPLEMENTATION

Now, I would like to hear about the process you went through to implement *TeleMOVE* Will you please describe how *TeleMOVE* was implemented at your site, to the best of your recollection? If you can, please tell me about the major milestones and the month and year in which they each occurred.

NOTE: Use any background information that might be available in terms of milestone dates, etc.

PROBES

- To what degree do you feel the Prescribed Implementation Activities have been implemented?

	Approximate Date Started-Completed	Not at all	Half Way	Fully Implemented
**Appropriate Roles Appointed (CCHT or MOVE!?)**				
**Get buy-in from staff**				
**Get buy-in from management**				
**Measure program outcomes**				
**Share information & best practices**				

NOTE: Table may be more useful for ER and DEG to fill out after the interview.

Will you please describe the process you used to enroll patients in *TeleMOVE*? Executing

PROBES

- How do patients get into the program?
- How do patients stay in the program?
- How does it end for them?

V. IMPLEMENTATION EXPERIENCES

Now, I'd like to ask some more specific questions about your experience getting *TeleMOVE* implemented and the type and level of support you had.

Can you describe the planning you did to get *TeleMOVE* implemented? Planning
PROBES

- Who was involved in the planning process? Were the appropriate people involved in the planning process? How engaged in the planning process were they?
- How did you track what tasks needed to be done? Progress? Status?

- What kind of marketing activities were done?
- Was there an evaluation component built in? How will you know if the program is successful or not?

Did you feel like you had enough flexibility or the ability to change aspects of *TeleMOVE* to implement it in a way that would work best at your site? Why or why not? Adaptability

PROBES

- What about the program was flexible? What was inflexible?
- How could *TeleMOVE!* be more flexible?
- How could *NCP and OTS* better accommodate unique differences at your site?

What level of involvement do leaders at your facility have with the *TeleMOVE* program? Leadership Engagement

PROBES

- Did they know about the *TeleMOVE! program*?
- What kind of support do they give you? Specific examples.
- Do they provide any feedback? What kind of feedback?
- Do you feel they are too involved or not involved enough? Why?

What kind of support did you receive from your VISN during implementation? Access to Knowledge and Information & Available Resources

PROBES

- From your facility?
- From NCP and OTS staff?
- Were the regular telephone calls with NCP or OTS helpful? Why or why not?
- Did you feel adequately supported to make the *TeleMOVE* work? Externally? Internally?

What type and how much IT support did you have when implementing *TeleMOVE*? Has this sustained over time? Access to Knowledge and Information & Available Resources

PROBES

- What were the most common technical issues you encountered? How were they resolved? (EMR support, Health Buddy device)
- Were you able to work with IT in order to allocate appropriate workload credit (stop codes)?

What about *TeleMOVE* Program materials distributed by NCP were helpful? Design Quality & Packaging

PROBES

- What was your experience in accessing the supporting materials for the *TeleMOVE* program? E.g., the *TeleMOVE* Implementation Guide; Patient Workbooks; Device; Scales.

Will you please describe the physical space configuration used for *TeleMOVE*? Available Resources

PROBES

- What kind of space was used for patient orientation and program enrollment? Group Visits?
- Where were you located (relative to others working on this DMP)?

Can you describe to me what is done with patient data once it is received? Executing

PROBES

- Do you find the data useful? Why or why not?
- Is the data used to make clinical decisions?

It sounds like you've worked really hard to get this *TeleMOVE* off the ground, what motivates you to do so? Individual Stage of Change

PROBES

- Will you be evaluated on how the program goes?

VI. COSTS

What kind of funding support did you receive for participating in *TeleMOVE*? Available Resources

PROBES

- FTE support? Staffing?
- Money?
- Relief from other duties?
- Were there resources or support that would have helped you implement *TeleMOVE*, that you didn't receive?

VII. PATIENT EXPERIENCE

What are your perceptions of the reasons patients choose to participate in the *TeleMOVE* program? Patient Needs & Resources

What is your impression of patients' early experience with the new *TeleMOVE* program?

VIII. LEVEL OF ENDORSEMENT AND RECOMMENDATIONS

On a scale of 0-10, how successful do you think piloting *TeleMOVE* at your site went? Why did you choose that number? Knowledge & Beliefs about the Intervention

Note: We are interested in the "perception" of success; the interviewee can define success in any way.

Based on your early experiences with *TeleMOVE* would you recommend continuing the program at your site? Why or not?

PROBES

- What would it take to keep *TeleMOVE* going at your site?
- What type of justification would you need to show in order to keep this intervention going over the long-term?

Would you recommend *TeleMOVE* to other sites? Why or why not?

Do you have any specific suggestions for other sites that have not yet started implementing *TeleMOVE*?

That's all the questions I have for you. Is there anything I've missed or anything you'd like to add?

IX. CONTACT INFORMATION

Before we sign off, I'd like to confirm your mailing information so we can get the $10 XY gift card out to you:
