# Supplementary material for: The Phased Implementation of a National Telehealth Weight Management Program for Veterans: Mixed-Methods Program Evaluation
Source: JMIR Diabetes. 2018 Oct 9;3(4):e14. doi: 10.2196/diabetes.9867 (PMC6307696; doi:10.2196/diabetes.9867)
Supplement: Multimedia Appendix 2 [file diabetes_v3i4e14_app2.pdf]

Appendix 2: Site Visit Follow Up Interview Guide
Evaluation of *TeleMOVE* Program Implementation

I. INTRODUCTION

Introduce self and other team members in the room

Hello, my name is [name of interviewer]. I am here with my colleague(s) [**name(s) of colleague(s)].** Thank you for agreeing to participate in this second follow up interview, we really appreciate your time.

Introduce the project

- As you know, the purpose of this CCHT-Weight Management study is to better understand barriers and facilitators to implementing the CCHT-WM disease manage program that your site encountered while piloting the program.
- We want to take this time to talk to you about your experience, so far, with the *CCHT-WM* program and learn more about the practical issues of delivering the program on a daily basis.
- For the purpose of this interview, we will refer to CCHT-WM as *TeleMOVE.*

Explain the purpose of the interview

- Your interview will help us to better understand the challenges and successes of implementing the *TeleMOVE* program at facilities similar to your site. We will be interviewing multiple people at your facility to gain multiple perspectives. We are really interested in learning more about your own experience with this program.

Describe the audio recording and how we will assure confidentiality and answer any questions

- This interview will be audio taped.
- Please be assured that the tapes and your transcript will be kept confidential. Leadership at your facility, any other co-workers, or NCP or OTS leadership will not have access to any of your responses nor be able to connect your responses to you personally. Only members of our research team will hear the interviews and the audio recording will be destroyed as soon as the transcript is verified and analyzed by the research staff.
- If, at any time, you feel that the questions are too sensitive, I would be happy to turn off the recorder during that portion of questioning. You may also skip any questions you wish during the interview.
- We also wanted to remind you, that we will be giving you a $10 ABC gift card as a small token of our appreciation for your participation.

Do you have any questions for me? [Answer any questions]

Are you ready to begin? I'm going to start recording now.

Appendix 2: Site Visit Follow Up Interview Guide
Evaluation of *TeleMOVE* Program Implementation

II. OPERATIONAL EXPERIENCES

In our last interview, we talked a lot about your experience in getting the program implemented. In this interview, I'm going to ask you about your experiences with the *TeleMOVE* program once it got up and running and how you feel it is functioning now.

First, I'm wondering if we can walk me through a typical week with your duties related to *TeleMOVE*.

PROBE

- How does *TeleMOVE* fit in with all of your duties?
- Do you feel that you have enough time to dedicate to fulfilling your responsibilities within the scope of the *TeleMOVE* program?
  - *If not, what aspect of your responsibilities for the program does this affect?*
- Are there other clinical priorities that compete for your time over *TeleMOVE*? If so… Can you tell me more about these priorities??

Could you please walk me through the process of how patients are enrolled in *TeleMOVE*?

PROBE

- How are patients screened for BMI at your facility?
  - Who determines which patients are eligible for MOVE!?
  - Who are major sources of referrals to MOVE!? To TeleMOVE?
- How many patients are typically referred to MOVE! for weight management?
- Who typically discusses MOVE! treatment options, including the *TeleMOVE* DMP?
- Do you sense that patients are aware of the existence of *TeleMOVE* at your facility?

Do you have a sense that patients enrolled in *TeleMOVE* are similar or different than patients enrolled in MOVE! or other wellness programming at your facility? If so, how so?

PROBE

- Are you aware what percentage of patients who decline?
  - Is the recruitment and enrollment information tracked and analyzed periodically?
- Who is responsible for enrolling patients?
- Are there certain personal characteristics that seem to define patients who sign up for *TeleMOVE*? (e.g., work during day, live far from VAMC, high BMI, ethnicity)

According to our first round of interviews, your facility's *TeleMOVE* is primarily run by _____ personnel (*insert* CCHT or MOVE!)?

Would you mind taking a couple minutes to help me understand how the program is run on a day-to-day basis?

PROBE

- How do patients get started in the program?
  - Is there an orientation session?
- What have patients' experiences been obtaining the proper equipment to begin the program (e.g., digital scales, patient workbooks, the DMP device)
- Have patients encountered any problems installing their device. If yes, tell me more…

- Who is responsible for tracking patients' progress?
- Who does the telephone follow-up calls? How are calls documented?
  - How many patients are each staff member expected to monitor?
- Do you have adequate time to monitor patients' progress on a regular basis?
- Are you using clinical reminders?
- Do you have a MOVE! related progress note title in the list of titles?

- How do you respond to red flag alerts?
- Do you find the red flags to be useful?
- Is there record keeping?
- Have you established clinic profiles for MOVE! related appointments?
- What are the biggest barriers to the program running smoothly?

How do you know the program is working or how do you know if the program is a success?
PROBES
- How do you track or measure patient progress?
  - Weight loss? Satisfaction? Hospital utilization? Other outcomes?
  - Number of patients who do more than one cycle of the DMP?
- Do you have time to track it or is it just another collateral duty that you're trying to accomplish amongst many other duties?
- Do you receive feedback from leadership about the program's success or about performance monitors?

<u>III. NETWORKS AND COMMUNICATION</u>
Now, I will ask you about the communication that takes place in order for *TeleMOVE* to function at your site.

What other units/services do you work with to implement *TeleMOVE*? (CCHT/MOVE!/Primary Care/Nutrition and Food Services)
PROBE
- How would you characterize your communication with these units/services?
- How is it working with Primary Care? Nutrition and Food Services?
- How do you handle red flags or patient requests for referrals to these services for problems encountered while using the DMP?

Some people rely more on formal communication (e.g., email communications through the chain-of-command) while others use informal communication (e.g., a hallway conversation with a co-worker) to accomplish things. Which type of communication has tended to be most helpful for you to accomplish your *TeleMOVE* related activities?
PROBE

- Focus on informal communications that include unscheduled discussions between staff outside of formal meetings.
- Were the informal communications helpful?
  - If there was not much informal communication, why not? Was the lack of informal communication a detriment?
- Ask for examples related to *TeleMOVE*.
- What are the most common forms of communication (e.g. face-to-face, meetings, email, telephone, etc.)

IV. LEADERSHIP INVOLVEMENT

What level of involvement do leaders at your facility have with the *TeleMOVE* program?

PROBES

- What types of support did leadership at your facility allocate for *TeleMOVE* implementation? Elicit specific examples.
- Did you think that they are familiar with the *TeleMOVE* program?
- What do you perceive to be the real priorities of leadership here? What is viewed to be important, what is not as important?
  - Do you think *TeleMOVE* meets a need for your facility?
  - Do you feel that *TeleMOVE* was compatible with your facilities processes of care?
  - With its values and beliefs regarding weight management?
- Do you receive any kind of goals or feedback from your facility's leadership about the achievements of *TeleMOVE*? If so, what kind?
- Do you feel like these leaders are too involved or not involved enough?

V. PATIENT EXPERIENCE

What is your impression of patients' early experience with the new *TeleMOVE* Program?

PROBES

- What has been the feedback you have heard from patients?
- What are the barriers for them in enrolling or participating in the program?
- Appeal of the *TeleMOVE* Program? (as opposed to regular MOVE!?)
- Appeal of doing program from home?
- What were their goals?

What did patients think of the MOVE! program? The *TeleMOVE* program?

PROBES

- What kind of impact did *TeleMOVE* have on patients?
  1. Opportunity to improve their health?
  2. Motivation to improve their diet or eating habits?
  3. Motivation to exercise more?

iv. Get extra attention?

- What expectations did patients have in participating in *TeleMOVE?*
- What is unique about patients at your site?
- What is the difference between people who choose MOVE! over *TeleMOVE*?
- What are the characteristics of people who choose to enroll?
- How difficult was it for patients to comply with goals?
- Was age a factor in how patients perceived the program? E.g. under 35?
- Was gender a factor in how patients perceived the program?
- Is gender a factor in how patients perceived the program?
- Is age a factor in how patients perceived the program? E.g. under 35?

VI. RESOURCES

What were the biggest resource constraints you encountered implementing the *TeleMOVE* program? (e.g. allocation of time, FTE, etc.)

How do you like the current device that is being used?

PROBES

- Are there any issues with getting the device set up? Patient user error?
- How does it compare to other Telehealth devices you've used/use?
- Do you like the way the data is tracked with this device?

Did you feel like you were adequately supported to deliver *TeleMOVE* well?

- Did you receive adequate training to prepare you to deliver *TeleMOVE?*
- What did you think of the online training and certification for delivering the program?
- Did you have access to the *TeleMOVE* Implementation guide?
  - If yes → Was it helpful? Why or why not.
- Did you feel like you had adequate access to information and resources to help you deliver *TeleMOVE* effectively to support patient weight loss goals?
  - If no → What would you need to feel more confident prepared or knowledgeable?

If I could wave a magic wand and give you everything you want or need, what would it take to get this program running better?

PROBES

- What resources would be needed to keep the *TeleMOVE* program going on a long-term basis?

One final question: How would you improve the *TeleMOVE* program if you could change any aspect of the program?

Thank you for participating in this interview. We hope the findings from this rapid response can be disseminated to other sites adopting *TeleMOVE* to help them get the program up and running as smoothly and effectively as possible. Your input into this report is very important.

Appendix 2: Site Visit Follow Up Interview Guide
Evaluation of *TeleMOVE* Program Implementation

I'm going to turn off the tape record but before I do, are there any questions you want me to answer about the study?
