# Supplementary material for: The Phased Implementation of a National Telehealth Weight Management Program for Veterans: Mixed-Methods Program Evaluation
Source: JMIR Diabetes. 2018 Oct 9;3(4):e14. doi: 10.2196/diabetes.9867 (PMC6307696; doi:10.2196/diabetes.9867)
Supplement: Multimedia Appendix 3 [file diabetes_v3i4e14_app3.pdf]

Multimedia Appendix 4. Site-level Indicators of *TeleMOVE* implementation over 2 years.

**Demonstration Site**	**1**	**2**	**3**	**4**	**5**	**6**	**7** [a]	**8** [a]	**9** [a]
**FY 10 Site Characteristics**									
Patients served per facility[b]	25,000	50,000	50,000	50,000	25,000	100,000	50,000	75,000	50,000
% patients aged ≥ 55 yrs	76%	74%	71%	76%	76%	82%	73%	74%	74%
% patients BMI ≥ 30	39%	36%	36%	40%	40%	36%	40%	40%	
% female patients	7%	9%	9%	7%	7%	7%	7%	9%	8%
% patients with rural residence	34%	67%	11%	68%	69%	19%	77%	64%	60%
Medical school affiliation Y/N	Yes	Yes	Yes	Yes	Yes	Yes	Yes	Yes	Yes
**Phase 2 Pilot Phase**									
Phase 2 patients enrolled[c]	23	49	2	23	60	32	6	19	0
Enrolled ≥ 30 patients? Y/N	No	Yes	No	No	Yes	Yes	No	No	No
**Year 1 Implementation**									
No. FY 2010 patients enrolled	27	90	34	122	147	52	11	47	0
Enrolled ≥ 100 patients? Y/N	No	No	No	Yes	Yes	No	No	No	No
Mean (SD) FY 10 weight change per patient, lb[d]	8.5 (10.3)	-.4 (9.8)	4.5 (10.6)	6.7 (15.1)	5 (14.3)	4.4 (16.9)	16.9 (14.8)	5.5 (16.4)	0 N/A
Mean weight loss? Y/N	Yes	No	Yes	Yes	Yes	Yes	Yes	Yes	No
Implementation uptake rating[e]	Low	Low	Low	High	High	Low	Low	Low	Low
Total site FY 10 weight loss, lb[f]	187	-22[b]	68	757	635	150	169	231	0
**Year 2 Implementation**									
No. FY 2011 patients enrolled	11	165	62	90	150	213	103	51	39
Enrolled ≥ 100 patients? Y/N	No	Yes	No	No	Yes	Yes	Yes	No	No
Mean FY 11 (SD) weight change per patient, lb[d]	1.9 (12.8)	0.3 (11.6)	6.1 (14.7)	7.1 (11.8)	6.2 (11.9)	5.2 (11.9)	8.7 (14.3)	11.3 (14.8)	-0.8 (7.4)
Mean weight loss? Y/N	Yes	No	Yes	Yes	Yes	Yes	Yes	Yes	No
Implementation uptake rating[e]	Low	Low	Low	High	High	Delayed High	Delayed High	Low	Low
Total site FY 11 weight loss, lb[f]	5.7	-11.8	375	549	611	645	608	383	-17.7

[a]Local staff declined to be interviewed.

[b]Totals for number of unique patients served rounded to nearest 25,000 patients served to preserve facility anonymity.

[c]Cumulative enrollment as of the end of Phase 2, February 28, 2010.

[d]Negative results indicate average weight gain for a site.

[e]Implementation uptake indicator calculated based on meeting enrollment target and attaining average weight loss, or high uptake whereas not achieving both of these indicators equated to low uptake.

[f]Total site weight loss is a function of total cumulative enrollment per year x mean clinical weight loss/gain for program participants.
