# Supplementary material for: The Phased Implementation of a National Telehealth Weight Management Program for Veterans: Mixed-Methods Program Evaluation
Source: JMIR Diabetes. 2018 Oct 9;3(4):e14. doi: 10.2196/diabetes.9867 (PMC6307696; doi:10.2196/diabetes.9867)
Supplement: Multimedia Appendix 4 [file diabetes_v3i4e14_app4.pdf]

Appendix 3. Summary of Formative Outcomes

Dates	Data Source	Formative process findings
**Phase 1**	1 VHA medical center	Sept – Nov 2009
	Demo site call notes	• Actual enrollment (n=27) vs. goal of 30-60 patients<br>• Finalize workload capture details for clinics, providers<br>• Group visits for orientation found most efficient
**Phase 2**	9 VHA Medical Centers	Oct 2009 – Feb 2010
	Call notes	• Understaffing slowed down enrollment
	Call notes/interviews	• Difficulty in changing vendor devices
	Call notes/interviews	• Clarify eligibility (need power, telephone land line, 8th grade literacy)
	Interviews	• Many patients have cell phones new digital landlines
	Call notes	• Dealing with patient vacations, interruptions in care
	Call notes/interviews	• NCP needs to offer monthly technical assistance calls
	Call notes	• Panel size reduced to 110--120 patients/coordinator
	Call notes	• Negotiate service agreement between MOVE!/HT staff
	Call notes/interviews	• Sites had issues switching to preferred vendor device
	Call notes	• Standardize training regardless of discipline
	Call notes/interviews	• Implementation guide/toolkit explains how to order program equipment, materials, and how to set-up EMR templates
**Phase 3**	Interested facilities	May 2010 - Sept 2011
	Interviews	• *TeleMOVE* is more time intensive/patient than MOVE
	Interviews	• In-person orientation is more efficient than by phone
	Interviews	• Enrollment and device installation are too logistically and technically complex
	Outcome analyses	• *TeleMOVE* weight loss is equal to standard MOVE!
	Interviews	• Facility and mid-level manager support is essential to overcome challenges, innovate, and sustain program
	Interviews	• Motivational calls by coordinators engage patients
	Interviews	• Sites with good cross-disciplinary communication and goal focus saw better implementation outcomes
	Interviews	• Regional learning networks/calls drive innovation, accountability, and knowledge sharing
	Interviews	• Cannot document type of patient encounter in EMR
	Interviews	• Vendor platform data does not interface with VHA databases
	Interviews	• DMP should be standardized across vendor devices
	Interviews	• Increase program reach and simplify logistics by converting to interactive voice response, smart phone or internet technology
	Interviews	• Empower clinicians with real time feedback on clinical outcomes in EMR or via facility-level reports
	Interviews	• Improve vendor database for quality improvement efforts (monitor patient-reported weight change, satisfaction, or red flag events)

NCP= National Center for Chronic Disease Prevention; TS = VA Telehealth Services; HT = Home Telehealth program; VAMC = VA medical center; DMP = disease management protocol; EMR = electronic medical record
